# Niosomal Benzoyl Peroxide and Clindamycin Lotion Versus Niosomal Clindamycin Lotion in Treatment of Acne Vulgaris: A Randomized Clinical Trial

**DOI:** 10.15171/apb.2019.066

**Published:** 2019-10-24

**Authors:** Saman Mohammadi, Abbas Pardakhty, Maryam Khalili, Reza Fathi, Maryam Rezaeizadeh, Saeedeh Farajzadeh, Azadeh Mohebbi, Mahin Aflatoonian

**Affiliations:** ^1^Department of Dermatology, Afzalipour Hospital, Kerman University of Medical Sciences, Kerman, Iran.; ^2^Pharmaceutics Research Center, Neuropharmacology Institute, Kerman University of Medical Sciences, Kerman, Iran.; ^3^Leishmaniasis Research Center, Afzalipour Hospital, Kerman University of Medical Sciences, Iran.

**Keywords:** Acne, Benzoyl peroxide, Clindamycin, Niosome

## Abstract

***Purpose:*** Combination of benzoyl peroxide (BPO) with topical antibiotics can lead to higher efficacy and less bacterial resistance, but it in turn increases adverse effects such as skin irritability and dryness. In this study, the efficacy of combination therapy of niosomal BPO 1% and clindamycin (CL) 1% is compared with niosomal CL in acne vulgaris.

***Methods:*** This is a double-blind clinical trial study on 100 patients with acne vulgaris in Afzalipour hospital in Kerman. Patients were randomly divided into 2 groups (case and control). The case group received niosomal combination of BPO 1% and CL 1%.The control group received niosomal CL1%. The efficacy of treatment protocols was evaluated in 2nd, 4th, 8th and 12th weeks of treatment by counting lesions (severity and grading acne lesions) and quality of life (QoL). Furthermore, side effect were evaluated at each treatment visits.

***Results:*** The reduction in mean percentage of acne lesions in case group (treated with BPO 1% and CL1%) (64.21%) was higher than control group (treated with niosomal CL 1%) (59.04%), but the statistical difference was not significant. Sum of excellent and good results were found in 80% and 76.1% of case and control groups, respectively (P=0.377). Also adding BPO to the treatment formulation in case group did not increase adverse effects, as statistical difference between 2 groups was not significant.

***Conclusion:*** Combination of niosomal BPO 1% and CL 1% in treatment of acne vulgaris showed higher efficacy with no increase in adverse effects in comparison with niosomal CL 1%, but the statistical difference was not significant.

## Introduction

Acne is a chronic inflammatory disorder of sebaceous unit that affects most frequently adolescents and young adult.^1^ Obstruction of follicular ducts due to impacted sebaceous and keratin secretions in accompaniment with *Propionibacterium acnes* colonization contribute to the pathogenesis of acne vulgaris.^2^ Different types of drugs such as keratolytic, antibacterial and retinoid can be used in treatment of acne. Topical antibiotics including erythromycin, clindamycin (CL) and azelaic acid have bactericidal and anti-inflammatory effects.^3^ CL is a lincosamide antibiotic that inhibits bacterial protein synthesis. Furthermore, CL reduces production of free fatty acids (by inhibition of lipase) and inhibits chemotaxis of leukocytes and pro-inflammatory cytokines. Using such drugs as monotherapy for more than 3 months or as maintenance is not recommended due to probable bacterial resistance.^4,5^

Benzoyl peroxide (BPO) has lipophilic properties (leading to better penetration through stratum corneum), keratolytic and bactericidal effects.^6-8^ Combination of BPO with topical antibiotics can lead to higher efficacy and less bacterial resistance at the cost of more side effects such as local irritability, peeling, dryness and burning sensation that is dose dependent.^9-11^

Niosomes are new drug delivery systems (NDDSs) with submicron particle size that can easily fuse in stratum corneum or pass through intra-epidermal channels and selectively affect target organ.In addition, more thermal stability and less oxidation capability of non-ionic surfactants in comparison to phospholipids (main constituents of liposomes) makes niosomes more favorable NDDS for topical delivery of active pharmaceutical ingredients. Based on our knowledge this is the first clinical trial of using combination of niosomal CL and BPO.^12-14^ Regarding to absence of niosomal combination formulation of BPO plus CL as pharmaceutical product in Iran drug market, the efficacy of combination therapy of niosomal BPO 1% and CL 1% was decided to be compared with niosomal CL 1% in the treatment of mild to moderate acne vulgaris.

## Materials and Methods

In this double-blind clinical trial study, 110 patients from Afzalipour hospital in Kerman were enrolled in the study, but finally 100 patients (50 patients in each group) completed the study. Inclusion criteria consists of patients aging from12 to 30 years old. Exclusion criteria consist of pregnancy, lactation, history of allergy to CL or BPO, patient with history of inflammatory bowel disease, colitis, polycystic ovary syndrome, hirsutism and patient taking neuromuscular blockers or oral anti-acne drug since 6 months ago and topical anti-acne drugs since 1 month ago. After signing the informed consent form, demographic features of patients (age and sex), clinical characteristics of lesions (location, grading and severity of acne) and quality of life (QoL) of participants were recorded. Then patients were instructed to apply topical formulation on cleaned and dried face twice daily for 12 weeks. Patients were divided by simple randomization with Minitab 16 (Mini Tab Inc.) in 2 groups (case and control) who received niosomal combination of BPO 1% and CL 1% and niosomal CL 1%, respectively. Drugs were preserved in similar bottle glasses, so none of the patients and the evaluator were aware of contents of bottles. Patients were instructed to apply sunscreen cream with sun protective factor of 30 every 2 hours on face as well. In order to evaluate patients’ compliance, they were instructed to return the empty bottle of drugs in the follow up visits.

The efficacy of treatment was evaluated in 2nd, 4th, 8th and 12th weeks of treatment protocols by counting of lesions, severity and grading acne lesions and QoL. Furthermore, side effects including erythema, scaling and pruritus were evaluated at each treatment visits.

The response to treatment was classified into 4 groups based on counting of the lesions: excellent (more than 75% reduction in acne lesions), good (between 51% to 75% reduction in acne lesions), fair (between 26% to 50% reduction in acne lesions) and poor (less than 25% reduction in acne lesions). In order to calculate the severity and grading acne lesions, Global Acne Grading System (GAGS) was used.^1,5^ According to GAGS, each site including forehead, each cheek, nose and chin are scored as 2, 2, 1 and 1, respectively. Also comedone, papule, pustule and nodules are scored as 1, 2, 3 and 4, respectively. The score of each site is calculated by multiplying the highest score of acne lesions by site score, and total score is the sum of 5 sites.

For evaluation of QoL, Persian version of Cardiff Acne Disability Index (CADI) questionnaire was used (reliability and validity was confirmed with Cronbach’s alpha coefficient = 0.79, Pearson correlation coefficient = 0.72).^1,6^ The questionnaire contains 5 questions about effects of acne on mood, social relationship, type of dressing , feelings and perceptions of patients about themselves. Each question has 4 choices scoring from 0 to 3. The final score is the sum of scores all questions. The lowest score represents the worst QoL.

### Niosomepreparation technique

Non-ionic surfactant vesicles were prepared by film hydration method.^1,7^ Briefly, lipid phase containing nonionic surfactant/cholesterol (7/3 molar ratio) was dissolved in 5 mL of chloroform and the organic solvent was eliminated by using rotary evaporator (Büchi Labortechnik AG, Switzerland) at 65°C. Thin lipid film was hydrated with 5 ml CL solution (1% w/v in phosphate buffered saline, pH 6.8) at 65°C for 30 minutes. Niosomal suspensions were kept in glass type I vials for further studies at refrigerator temperature. For BPO lipid vesicles, similar method was used, but BPO and lipids were dissolved in ethanol 96/chloroform (50/50 v/v) and the final concentration of BPO in niosomal formulations was 1% w/v.

### CL and BPO concentrations measurement

Ultraviolet (UV) first derivative spectrophotometric method has been developed for determination of CL in free and niosomal formulations at 251 nm with linearity range 60-200 µg/mL in deionized water. BPO was dissolved in ethanol 96 and UV spectrophotometry was used at 244 nm for its concentration measurement.

### Encapsulation efficiency determination

To determine the amount of the drug, 1 mL of isopropyl alcohol was added to the pellet to dissolve the walls of the niosomes and then the UV-visible spectrophotometer was determined. In the case of CL, the second derivative of the UV spectrum was used.

### CL and BPO release study

For release study of CL and BPO from niosomes, all glass Franz diffusion cell was used. The used diffusion cell had a receptor compartment volume of about 37 mL. Then, 1 mL of the product was placed on the cellophane membrane. To conduct the delivery test, the cellophane membrane was first submerged in the recipient phase for 24 hours to maintain a membrane thickness during the test. During the test, the temperature of the enclosure around the receiver phase was kept constant by a circulating water at 37 ± 1°C. In order to prevent the accumulation of active material released, in other words, for uniform distribution of the active substance in the receiver phase and uniform distribution of the temperature, a magnetic stirrer was used at medium speed (100 ± 5 rpm). Sampling from receiver phase was performed at 0, 15, 30, 60, 90, 120, 180, 240 min at sink conditions.

### Statistical analysis

For descriptive analysis, we used frequency, relative frequency and mean ± standard deviation. For comparison of quantitative variables between 2 groups (age, sex, number of lesions, duration of the disease, QoL and GAGS) *t* independent test was used. To compare treatment efficacy and adverse effects between the 2 groups, chi-square test and Fisher exact test were used. The sample size was calculated 100 with statistical power of 80%, based upon the findings of a pilot study with percentage sum of good and excellent efficacy in case and control groups (62% and 32% respectively).

## Results

**Figure 1:**
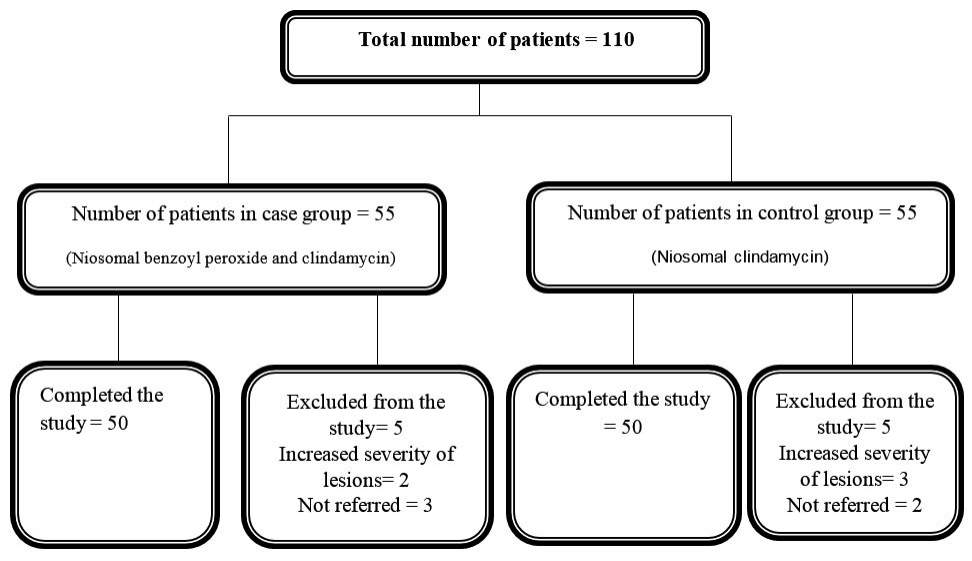
Study algorithm.

In this study, 100 patients (50 patients in each group) with mild to moderate acne vulgaris completed the study (Figure 1). Most of the patients were female (82%) and mean age of the patients (aging from 13 to 30) was 18.64 ± 3.32. Duration of acne in case and control group was 2.30 ± 0.16 and 6.62 ± 0.28 years, respectively (*P* = 0.339) (Table 1).

**Table 1:** Base line and demographic characteristics in both treatment groups

	**Niosomalclindamycin**	**Niosomalclindamycin/benzoyl peroxide**	***P*** **value**
Age (y), mean ± SD	19.54 ± 0.73	18.64 ± 0.47	0.378
Sex, No. (%)			
Male	8 (16)	10 (20)	0.603
Female	42 (84)	40 (80)	
Lesion counts, mean± SD			
Non-inflammatory	16.10 ± 2.12	18.02 ± 1.86	0.498
Inflammatory	14.45 ± 1.73	10.14 ± 1.02	0.031
Total	30.56 ± 2.76	28.16 ± 2.15	0.491
Duration of disease (y)	18.92 ± 0.67	16.34 ± 0.43	0.339
CADI score	11.38 ± 1.63	11.89 ± 1.77	0.146

### Assessment based on counting of acne lesions

**Figure 2:**
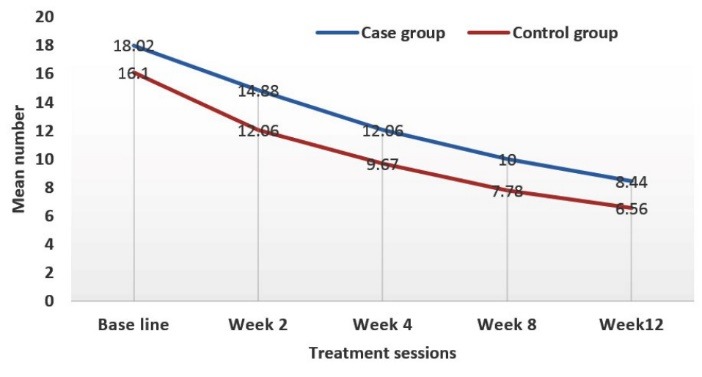
Mean number of non-inflammatory lesions during treatment sessions.

**Figure 3:**
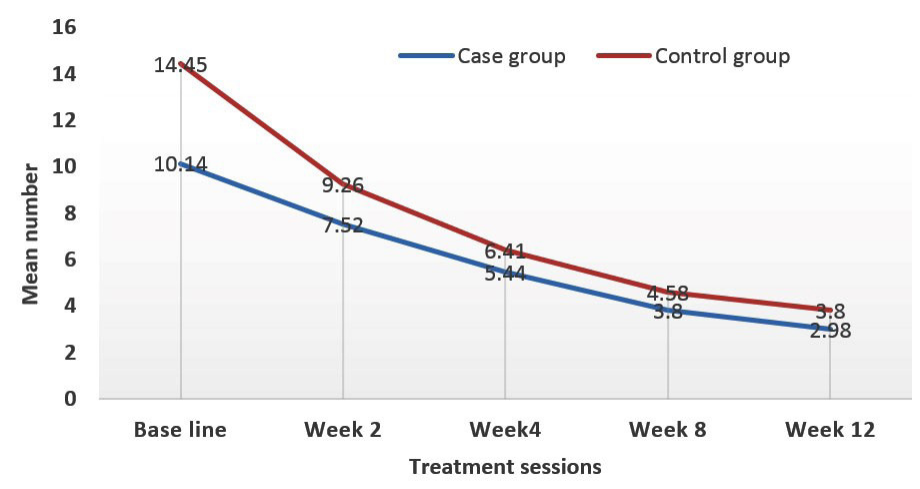
Mean number of inflammatory lesions during treatment sessions.

**Figure 4:**
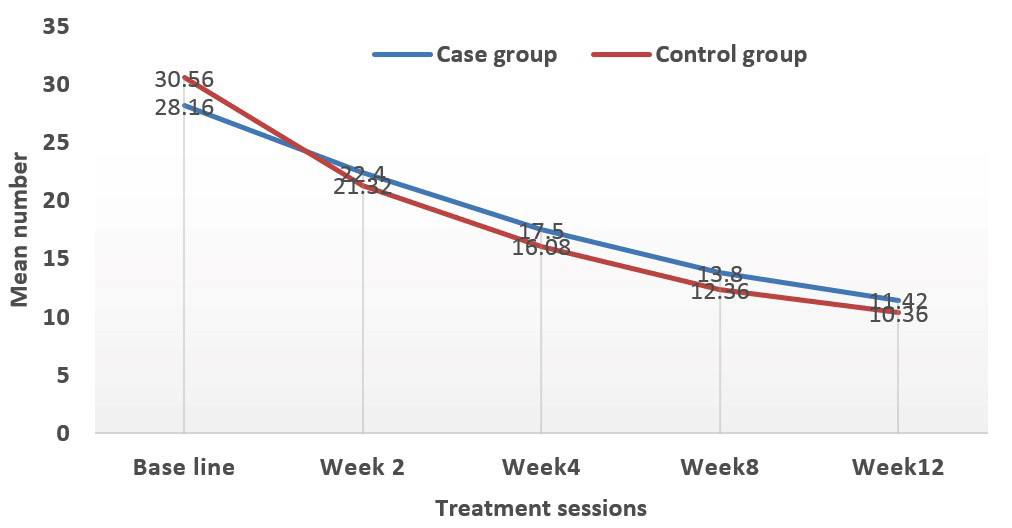
Mean number of total lesions during treatment sessions.

At the beginning of the study, mean number of total acne lesions in case and control groups was 28.16 ± 2.15 and 30.56 ± 2.76, respectively (*P* value = 0.49). At the end of the study, these were 11.42 ± 0.91 and 10.36 ± 1.03, respectively (*P* value = 0.44) (Figures 2-4).

### Assessment based on GAGS score

At the beginning of the study, GAGS score in case and control groups was 14 ± 0.90 and 13.94 ± 0.43 (*P* value = 0.95), respectively. At the end of the study, these were as 7.76 ± 0.63 and 6.64 ± 0.46, respectively (*P* value = 0.15) (Table 2).

**Table 2:** GAGS score in follow up visits in case and control group

**Treatment visits**	**Case group**	**Control group**	***P*** **Value**
Base line	14± 0.90	13.94± 0.43	0.95
2nd week	12.56± 0.83	10.74± 0.31	0.04
4th week	10.24± 0.75	9.28± 0.38	0.26
8th week	9± 0.70	7.96± 0.39	0.20
12th week	7.76± 0.63	6.64± 0.46	0.15

### Assessment based on percentage of reduction in acne lesions

At the end of the treatment, rates of excellent, good, fair and poor response in case group were 18%, 62%, 20% and 0%, respectively. These rates in control group were 28.3%, 47.8%, 21.7% and 2.2%, respectively (*P* = 0.37).

### Assessment based on QoL

The mean score of QoL in case group before and after the treatment was 11.89 ± 1.77 and 8.60 ± 2.52, respectively (*P* < 0.001). The mean score of QoL in control group before and after treatment were11.38 ± 1.63 and 10.06 ± 2.04, respectively (*P* < 0.001). Also, the statistical difference between 2 treatment groups at the end of the treatment was significant (P = 0.003).

### Adverse effects of treatment

Table 3 shows adverse effects of treatment in 2 groups of case and control. There was no significant difference between 2 groups regarding the adverse effects including erythema, pruritus and peeling.

**Table 3:** Side effects of treatment in both treatment groups during follow up visits

**Side effects**	**Treatment visits**	**Severity**	**A (%)**	**B (%)**	***P*** **value**
Erythema	2^nd^ week	Mild	7 (53.8)	6 (46.2)	0.68
		Moderate	2 (66.7)	1 (33.3)	
	4^th^ week	Mild	8 (57.1)	6 (42.9)	
	8^th^week	Mild	3 (50)	3 (50)	
	12^th^ week	Mild	2 (40)	3 (60)	
Pruritus	2^nd^ week	Mild	13 (52)	12 (48)	0.30
		Severe	0 (0)	1 (100)	
	4^th^ week	Mild	13 (52)	12 (48)	0.30
		Moderate	0 (0)	1 (100)	
	8^th^week	Mild	4 (40)	6 (60)	0.42
		Moderate	0 (0)	1 (100)	
	12^th^week	Mild	4 (40)	6 (60)	0.42
Scaling	2^nd^ week	Mild	15 (50)	15 (50)	
	4^th^ week	Mild	19 (48.7)	20 (51.3)	
	8^th^ week	Mild	16 (45.7)	19 (54.3)	
	12^th^ week	Mild	13 (43.3)	17 (56.7)	

Topical therapy is used as first-line of treatment in mild to moderate acne lesions based on the type and severity of the lesions (inflammatory and non-inflammatory), tolerability of patients, type of skin and comorbidities. Previous studies demonstrated that 90% of patients with acne were resistance to at least 1 topical antibiotic and efficacy of the most antibiotics decreased over time due to bacterial resistance. Nowadays, combination therapy with different mechanisms of action is preferred method of choice that leads to more response rate, lower adverse effects as well as easier application of drug.^18^

In the present study, the efficacy of combination therapy with niosomal BPO 1% and CL 1% versus niosomal CL 1% in acne vulgaris was evaluated. The percentage of improvement of acne lesions in the group treated with niosomal BPO 1% and CL 1% (64.21%) was found higher than niosomal CL 1% (59.04%), but the statistical difference was not significant.

In one study by Gold in 2009, mean percentage of reduction of non-inflammatory, inflammatory and total acne lesions with CL1.2% and BPO2.5% gel was estimated 48.7%, 64.1% and 52%, respectively (vs. 55.74%, 77.69% and 64.21% in our study) that was lower than the present study.^19^

In another study by Thiboutot and colleagues in 2008, mean rate of reduction of non-inflammatory, inflammatory and total acne lesions with CL 1.2% and BPO 2.5% was 43.2%, 54.6% and 47.9% ( vs. 55.74%, 77.69% and 64.21% in the present study) , respectively.Also, in Thiboutot study mean rate of reduction of non-inflammatory, inflammatory and total acne lesions with conventional CL solution was 36.2%, 46.2% and 40.4% respectively (vs. 47.5%, 71.63% and 59.04% in our study) that was far lower than the present study.^20^ Higher response rate to lower concentration of BPO in the current study (1%) than Gold and Thiboutot et al studies (2.5%) can be explained by niosomal formulation of BPO which leads to better delivery of drug to target organ.^19,20^

Modern systems of drug delivery such as niosomes have higher efficacy and lower side effects. Niosomes are characterized by vesicle structures with nanometer size composed of non-ionized surfactants and cholesterols. These structures have an advantage of easy transport through bilayer lipid structure of stratum corneum. Equal efficacy is achieved with lower doses of drug due to selective absorption in target organs and better penetration of niosomal structure as a result of small size and flexibility of structure.^12-14^ Gupta et al in 1 in vivo study demonstrated equal therapeutic index of niosomal formulation of BPO in comparison with conventional form using 4.16 time lower dosage.^21^

Today, there are few studies evaluating efficacy of niosomal formulations in acne. In 1 study in Kerman in 2017 by Mohammadi and colleagues, rate of good and excellent improvements with niosomal erythromycin solution was observed in 39.9% of patients that was lower than the present study in combination of BPO 1% and CL 1% (80%). Synergistic effect of BPO and CL due to different mechanisms of action, and lack of bacterial resistance to BPO could be due to higher efficacy in our study than Mohammadi’s study. Also, the rate of good and excellent improvements with niosomal erythromycin solution (39.9%) was lower than niosomal CL (76.1%).^22^ This can be explained by higher rate of bacterial resistance to erythromycin than CL which leads to lower relative efficacy of erythromycin in comparison to CL.^23^ In the present study both groups experienced mild adverse effects which was in concordance with Mohammadi’s study.^22^ Moreover, adverse effects such as erythema, peeling and pruritus were observed with more frequency and severity at first 4 weeks which was in agreement with Mohammadi’s study. During these course of treatments patients’ tolerance increased, so less severity and frequency of adverse effects was observed.^22^

In one study by Kawashima and colleagues in Japan, efficacy of combination therapy of CL 1.2% plus BPO 3% was compared with CL 1.2%. In this study, mean reduction in acne lesions was statistically significant in combination group than monotherapy with CL, but higher percentage of adverse effects was observed in combination group (35.1%) than monotherapy with CL (9%). In the present study, adverse effects were observed in 30% of the patients in combination group, but most of them were mild. Moderate and severe side effects were only seen in 2 (4%) of the patients of our study, but with higher rate in Kawashima et al study.^10^ This can be explained by lower concentration of BPO in the current study (1%) than Kawashima et al study (3%) and niosomal formulation of BPO in the present study.

In previous studies, it has been demonstrated that combination therapy BPO with CL can target different causes of pathogenicity in acne patients and lead to higher and faster response rate in treatment, but it increased adverse effects of treatment such as irritability (especially with higher concentration of BPO) which leads to poor adherence and compliance of the patients.^6-8^ Niosomal structures have the advantage of selective and gradual drug release in target organ (pilosebaceous unit) which leads to lower adverse effects and more efficacy of treatment as observed in our study.^14,15^ Furthermore, other advantages of this type of treatment are including more adherence of patients to treatment, rapid control of inflammation, lower percentage of post inflammatory pigmentation and scar formation that is especially observed in darker skin types.^24^

## Conclusion

This study shows more reduction in acne lesions in the group treated with niosomal BPO 1% and CL 1% than niosomal CL 1%, but the statistical difference between 2 groups was not significant. Adding BPO to formulation in case group didn’t lead to higher adverse effects, and no statistical difference between 2 groups was observed. So increased efficacy and less adverse effects is a major advantage of niosomal and combination formulations in treatment of acne lesions, especially in adolescents who have low compliance with higher expectations.

## Ethical Issues

This proposal was approved in ethical committee of Kerman University of Medical Sciences and registered with code IR.kmu.rec.1395.116.

## Conflict of Interest

Authors declare no conflict of interest in this study.

## Acknowledgments

This work was supported by the research department in Kerman University of Medical Sciences, Kerman, Iran.
